# Effects of Parecoxib and Fentanyl on nociception-induced cortical activity

**DOI:** 10.1186/1744-8069-6-3

**Published:** 2010-01-21

**Authors:** Yuan-Zhi Peng, Xiao-Xi Li, Ying-Wei Wang

**Affiliations:** 1 Department of Anesthesiology, Xinhua Hospital, Shanghai Jiaotong University School of Medicine, Shanghai 200092, China

## Abstract

**Background:**

Analgesics, including opioids and non-steroid anti-inflammatory drugs reduce postoperative pain. However, little is known about the quantitative effects of these drugs on cortical activity induced by nociceptive stimulation. The aim of the present study was to determine the neural activity in response to a nociceptive stimulus and to investigate the effects of fentanyl (an opioid agonist) and parecoxib (a selective cyclooxygenase-2 inhibitor) on this nociception-induced cortical activity evoked by tail pinch. Extracellular recordings (electroencephalogram and multi-unit signals) were performed in the area of the anterior cingulate cortex while intracellular recordings were made in the primary somatosensory cortex. The effects of parecoxib and fentanyl on induced cortical activity were compared.

**Results:**

Peripheral nociceptive stimulation in anesthetized rats produced an immediate electroencephalogram (EEG) desynchronization resembling the cortical arousal (low-amplitude, fast-wave activity), while the membrane potential switched into a persistent depolarization state. The induced cortical activity was abolished by fentanyl, and the fentanyl's effect was reversed by the opioid receptor antagonist, naloxone. Parecoxib, on the other hand, did not significantly affect the neural activity.

**Conclusion:**

Cortical activity was modulated by nociceptive stimulation in anesthetized rats. Fentanyl showed a strong inhibitory effect on the nociceptive-stimulus induced cortical activity while parecoxib had no significant effect.

## Background

Pain is a complex subjective sensory experience which is comprised of sensory discrimination, cognitive appreciation and affective motivation components [1]. The treatment of pain is a fundamental issue in the practice of anesthesia. However, the mechanisms of pain and analgesia are unclear. With the development of imaging technology (e.g., functional magnetic resonance imaging and positron emission tomography), cortical areas that are commonly activated by noxious stimuli have been illuminated. These areas include the primary and secondary somatosensory cortices (S1, S2), anterior cingulate cortex (ACC), insular cortex and motor cortex [2,3]. Electrophysiological studies show that cortical neuronal activity under anesthesia is associated with an Up-Down membrane potential fluctuation, while EEG signals are characterized by large-amplitude and slow-wave activity [4-6]. It is well known that synapses are highly plastic in the central nervous system including ACC, and that long-term changes in synaptic transmission contribute to different functions of brain including chronic pain. Long-term potentiation (LTP) is believed to the basis of learning and memory, and it can also be induced by different protocols or peripheral injury at the spinal cord dorsal horn [7,8]. Elucidation of the electrophysiological mechanisms of the neuron in encoding nociceptive information may provide new advances in the detection of pain under anesthesia. Despite the effectiveness of opioid drugs in postoperative pain management, there are limitations in their use, partly due to tolerance effects. In addition, opioid drugs can potentially cause respiratory depression, nausea and vomiting [9]. Non steroid anti-inflammatory drugs (NSAIDs) have been used in combination with opioid analgesics to reduce postoperative opioid administration [10,11]. Parecoxib, a selective inhibitor of cyclooxygenase-2 (COX-2), can block prostaglandin biosynthesis associated with inflammatory pain. Moreover, parecoxib lacks the potential adverse effects of COX-1 inhibition, including gastroduodenal ulceration and bleeding or impairment of platelet function [10,11]. However, it has been argued that parecoxib is lacking in adequate analgesic effects on postoperative pain [12].

The present study investigates the neuronal activity of ACC and S1 areas (two regions known for their involvement in pain processing) during noxious stimulation. In addition, the effect of parecoxib was compared with fentanyl on evoked cortical activity.

## Results

### Cortical response to nociceptive stimulation

**Figure 1:**
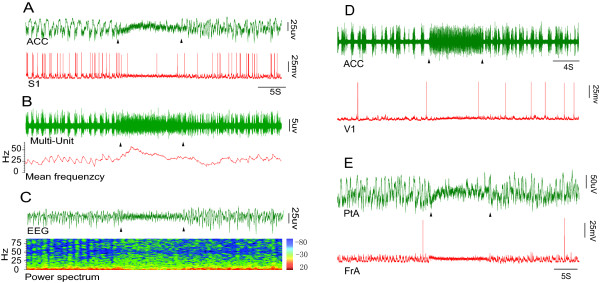
**Electrophysiological recordings from cortical areas during nociceptive stimulation**. A, upper panel: Trace of the extracellular signal from the ACC, showing that the amplitude was reduced while the frequency was increased when a nociceptive stimulus was applied; lower panel: Intracellular trace from S1, showing that the Down state disappeared and the membrane potential (V_m_) switched to a persistent Up state during nociceptive stimulation. B: The Multi-Unit signal was filtered from the upper panel in A. The spike firing rate of the MU signal was increased, and the Down state disappeared under nociceptive stimulation. C, upper panel: the trace filtered from A (upper panel), note that EEG waves switched to low-amplitude, high-frequency pattern activity; lower panel: The power spectrum of the corresponding EEG (C, upper panel), showing that the low frequency band was markedly reduced. D: The traces recorded from the ACC (upper panel) and from V1 (lower panel). E: The traces recorded from PtA (upper panel) and FrA (lower panel). Triangles signify the start and end of nociceptive stimulation. The color bar represents the proportion of EEG bands.

We simultaneously measured extracellular activity in the ACC and intracellular activity in the S1 region in response to nociceptive stimulation. We found that both the ACC and S1 areas were activated by tail pinch in urethane anesthetized rats (Fig. 1A). To examine whether the neural activity of other cortical areas could be altered by nociceptive stimulation, we performed recordings at V1(primary visual cortex), PtA(parietal association cortex) and FrA(frontal association cortex), which presented similar activity to that seen in ACC and S1 (Fig. 1D and 1E).

**Figure 2:**
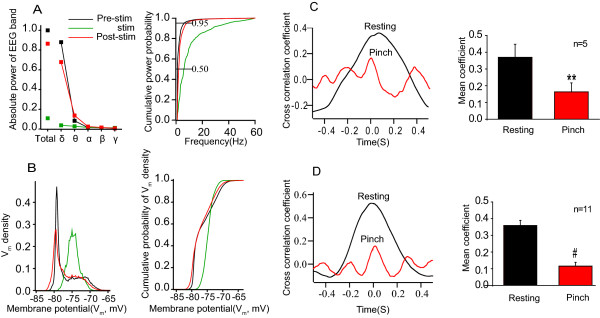
**Changes in EEG, Vm and waveform correlation during nociceptive stimulation**. A, left panel: Nociceptive stimulation reduced normalized total power of each EEG band; right panel: The cumulative power probability of the EEG, was shifted to the right, under nociceptive stimulation. B, left panel: The distribution of membrane potential (V_m_), was bimodally distributed under the resting condition, and unimodally distributed when nociceptive stimulation was applied; right panel: The cumulative probability of V_m _density is plotted before, after and during nocicptive stimulation. All measures returned to pre-stimulation values after nociceptive stimulation was removed. C and D: Waveform correlation between the ACC and S1 showing that the correlation coefficient decreased during tail pinch; in C extracellular recordings were recorded in both regions, in D intracellular recordings were recorded at S1 while extracellular recordings were recorded at ACC. The left panel in both C and D show a sample correlation coefficient before and during nociceptive stimulation. The right panel plots the group data. Each EEG power was normalized to the total power of Pre-stimulation values. The correlation coefficient (peak value near zero seconds) was considerably higher in the resting state than during tail pinch.

The EEG recordings showed a shift from large-amplitude, low-frequency waves (commonly seen in anaesthetized rats) towards low-amplitude, high-frequency waves (resembling that of awake rats) during tail pinch (Fig. 1A and 1C, upper panels). In addition, the mean firing rate obtained from multi-unit(MU) recordings was enhanced and the Down state disappeared (Fig. 1B). The power spectrum analysis revealed that during tail pinch the ratio of power within low frequency bands was markedly decreased (Fig. 1C), along with the decreased absolute power of each EEG band (Fig. 2A).

Intracellular recordings showed that the membrane potential (V_m_) exhibited a bimodal distribution with Up-Down transitions in the resting condition. This pattern quickly switched to a persistent Up state following nociceptive stimulation (Fig. 1A, D and 1E, lower panels; Fig. 2B). Furthermore, waveform correlation analysis revealed that highly synchronized activity was observed between the ACC and S1 recordings in the resting state, while desynchronization was presented when nociceptive stimulation was applied (Fig. 2C and 2D). The desynchronization was observed when comparing extracellular recordings in both the ACC and SI (Fig. 2C right panel, p < 0.01), and when comparing extracellular recordings in the ACC with intracellular recordings in SI (Fig. 2D, p < 0.001). Together, these results provide direct electrophysiological evidence for extensive alterations in cortical activity by nociceptive stimulation, as suggested by previous EEG and imaging studies [2,3,13].

### Effects of parecoxib and fentanyl on cortical activity

**Figure 3:**
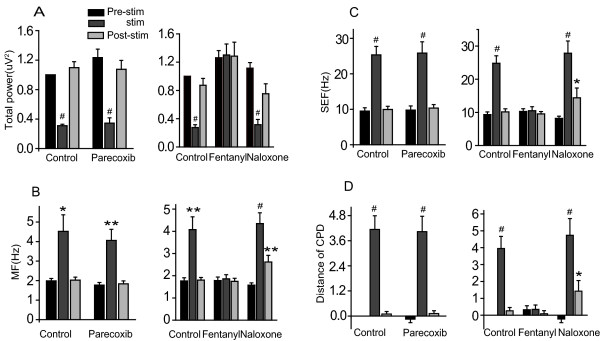
**The effect of parecoxib and fentanyl is evaluated by EEG parameters**. Total power (A) was decreased during nociceptive stimulation, while MF (B), SEF (C), and the distance of cumulative power distribution (CPD) increased in response to noxious stimulation(D). Fentanyl (n = 12) significantly inhibited the response in each case and the inhibition was reversed by naloxone (n = 9). Parecoxib had no effect on the neuronal response to noxious stimulation (n = 11). Total power and distance of cumulative power distribution was normalized to pre-stimulation values. *P < 0.05, **P < 0.01, #P < 0.001, compared with pre-stim value.

The effects of parecoxib and fentanyl on cortical activity in response to acute mechanical pain were compared. Extracellular recordings at ACC and intracellular recordings at S1 in urethane anesthetized rats were obtained. EEG recordings revealed that normalized total power (Fig. 3A) was decreased, while MF (medial frequency; see Fig. 3B), SEF (95% spectral edge frequency; see Fig. 3C) and distance of cumulative power distribution (Fig. 3D) were increased by nociceptive stimulation compared to the resting state. Fentanyl significantly inhibited these effects, and moreover, its action was reversed by naloxone (see Fig. 3). Parecoxib administration, on the other hand, did not inhibit the observed response in any of these measures (Fig. 3).

**Figure 4:**
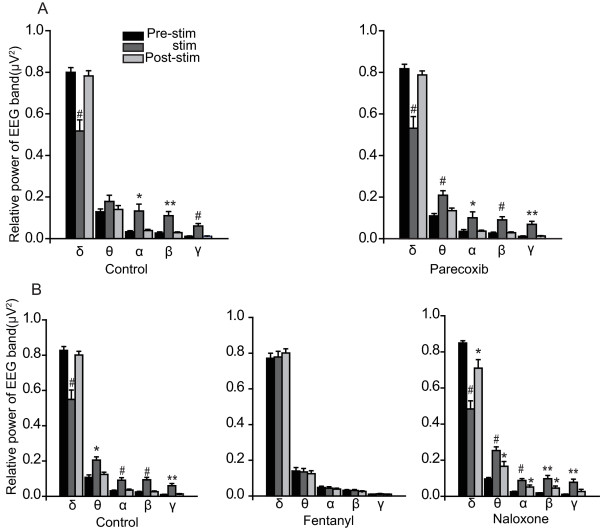
**Group data for relative power of each EEG band recorded from the ACC**. δ band power was decreased while other band powers were increased in response to nociceptive stimulation. A: Parecoxib had no effect on band power response. B: Fentanyl completely inhibited the band power response to nociceptive stimulation and naloxone reversed its effect. *P < 0.05, **P < 0.01, #P < 0.001.

There was a significant decrease in δ band power and an increase in other band powers when nociceptive stimulation was applied (Fig. 4A and 4B, control group), and a similar change was observed in the parecoxib group (Fig. 4A, right panel). However, there were no significant changes in any EEG band after fentanyl injected (fig 4B, middle panel). In addition, naloxone reversed the action of fentanyl (Fig. 4B, right panel).

**Figure 5:**
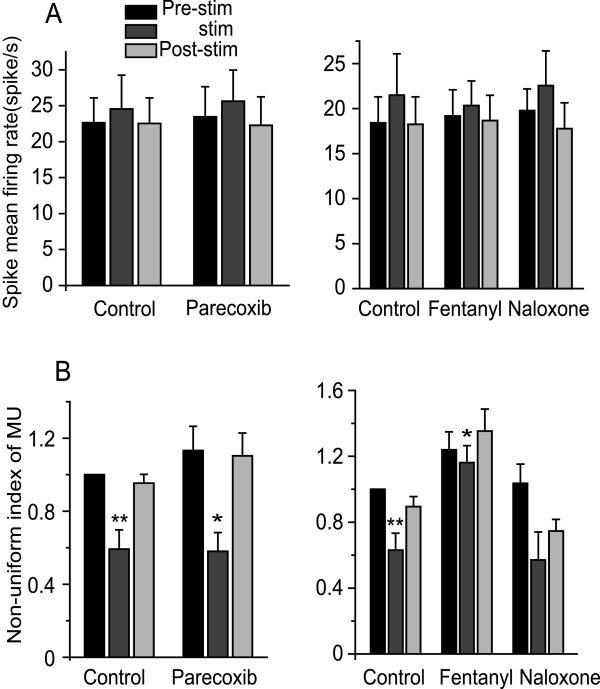
**The effects of parecoxib and fentanyl on MU (Multi-Unit) and Non-uniform index of MU recordings**. A: Spike mean firing rate from MU recordings were not altered in the presence of nociceptive stimulation. B: The non-uniform index of MU was decreased when nociceptive stimulus was applied (control), indicating that the neuronal firing pattern was more uniform under nociceptive stimulation than during the resting state. Fentanyl partially inhibited this response while parecoxib had no effect. *P < 0.05, **P < 0.01, #P < 0.001.

Although the Down state was reduced or disappeared (Fig. 1), nociceptive stimulation did not influence the spike mean firing rate from MU recordings (Fig. 5A). However, the firing rate was reduced in some neurons (Fig. 1A and 1E; lower panel), and this inhibition was also observed in previous studies [14,15]. We therefore, further investigated spike firing rate by a Non-uniform index of spike frequency to evaluate effects of parecoxib and fentanyl. This measure showed that nociceptive stimulation decreased the "non-uniform index" in control (untreated) animals and that parecoxib did not inhibit this response; The response was partially inhibited, however by fentanyl and again, naloxone abolished the fentanyl effect (Fig. 5B).

**Figure 6:**
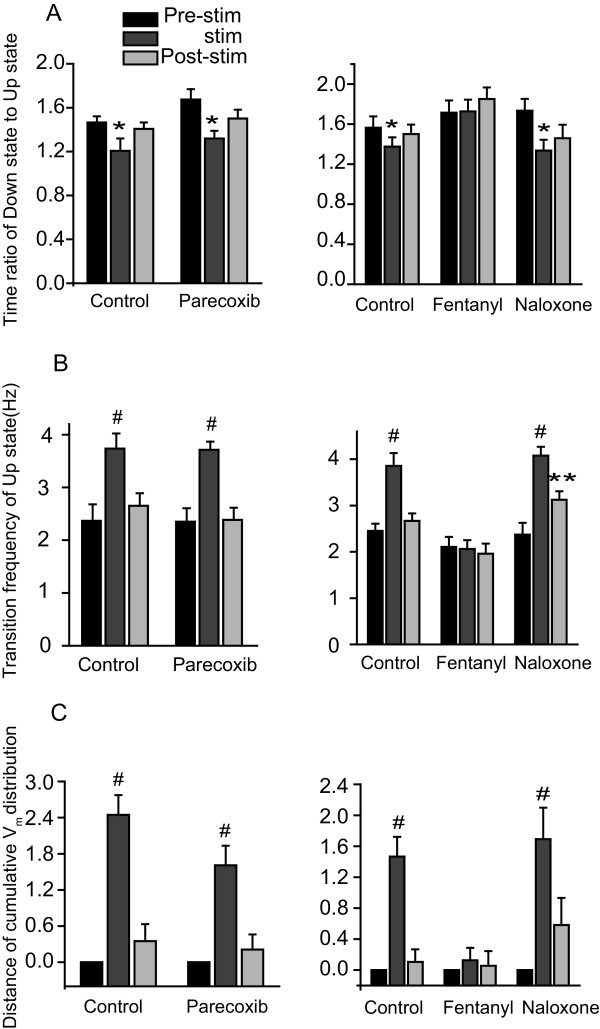
**Group data for intracellular recordings at S1**. The time ratio of Down state to Up state decreased (A, control), while transition frequency of Up state and the distance of cumulative V_m_distribution increased (B and C, control) in response to nociceptive stimulation. The response of all measures to nociceptive stimulation was not inhibited by parecoxib (left panels, n = 8). Fentanyl (right panels, n = 12) inhibited all responses and naloxone reversed fentanyl's action (n = 9). *P < 0.05, **P < 0.01, #P < 0.001.

Finally, we investigated the Up and Down states under different conditions in the S1 region. The ratio of time in the Down state to time in the Up state was reduced and the firing rate of the Up state increased (Fig. 6A, B; control) during nociceptive stimulation. Administration of fentanyl reversed these responses while the addition of naloxone abolished the fentanyl's effects (Fig. 6A and 6B, right panels). In addition, the distance of membrane potential distribution was significantly increased during tail pinch (Fig. 6C; control) and administration of fentanyl significantly inhibited this response. Naloxone was again able to reverse the fentanyl effect (Fig. 6C, right panel). Parecoxib had no significant effect on any of these measures (Fig. 6, left panels).

**Figure 7:**
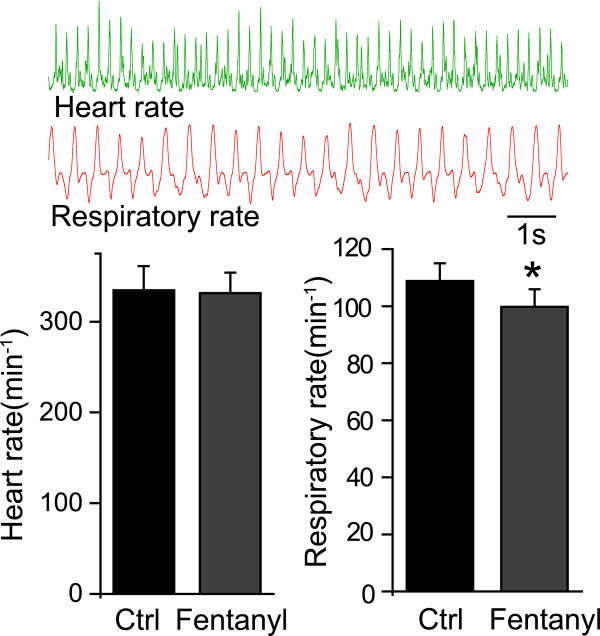
**The effect of fentanyl on heart rate and respiratory rate**. The two upper panels show examples of heart rate and respiratory rate recordings. The left, lower graph shows that fentanyl did not alter heart rate. The right, lower graph shows that fentanyl had a small but significant effect on respiratory rate. *P < 0.05.

Fentanyl (30 μg/kg, i.m.) did not significantly influence heart rate, while respiratory rate was lightly depressed (Fig. 7, n = 9). Further study showed that there was no significant change in the index of blood gas, glucose or hematocrit (see table 1).

**Table 1:** Blood gas, Glucose and Hemotacrit values

	Control	Fentanyl
pH	7.31 ± 0.02	7.28 ± 0.02
PaCO_2 _(mmHg)	43 ± 2	48 ± 3
PaO_2 _(mmHg)	98 ± 3	99 ± 4
SO_2_	96.8 ± 0.4	96.8 ± 0.6
Base excess (mmol/L)	-5.3 ± 0.9	-4.6 ± 0.9
Glucose (g/dL)	15.0 ± 0.5	14.2 ± 0.3
Hemotacrit (%)	48.2 ± 1.5	45.9 ± 1.1

## Discussion

The present study investigated the neuronal response in the ACC and S1 area of urethane anesthetized rats during acute mechanical noxious stimulation. In addition, the effects of parecoxib and fentanyl on evoked neuronal responses were assessed. Acute mechanical stimulation led to neuronal activation in all cortical areas studied. In a more detailed investigation using the ACC and S1 areas specifically, we found that peripheral nociceptive stimulation induced a transition from typical Up and Down state dynamics to a long-lasting EEG activated state. In addition, intracellular recordings showed that membrane potentials were locked into a persistent Up state during noxious stimulation resulting in a desynchronization among cortical brain areas. Fentanyl successfully inhibited the extracellular and intracellular responses induced by noxious stimulation and naloxone reversed the fentanyl' effects. Parecoxib, on the other hand, had no effect on neuronal response.

Previous studies have shown that EEG waves are desynchronized when a noxious stimulus is applied to a lightly anesthetized human or animal [13,16]. EEG desynchronization along with a significant decrease in total power and δ and θ band frequency have also been observed after noxious stimulation in isoflurane anesthetized goats [13]. Results from the present study show that all cortical areas investigated were activated by noxious stimulation (Fig. 1). This non-specificity in cortical activation may be restricted to anesthetized animals. Previous studies show that this type of total cortical activation can also be mimicked by stimulation of the ascending arousal system during anesthetic treatment [17,18]. For example, brief electrical stimulation of the pedunculopontine tegmentum (PPT) or locus coeruleus under ketamine-xylazine anesthesia typically induced a prolonged period of desynchronized EEG activity that was paralleled by a persistent depolarized state of the membrane potential. These conditions are similar to that observed in the natural awake state [17,18]. Our results are consistent with those observed with the PPT stimulation in that the membrane potential was locked into a persistent Up state when nociceptive stimulation was applied to the tail. These findings indicate that the ascending arousal system can be activated directly by nociceptive stimulation. In other words, if the nociceptive stimulation had only activated the spinothalamic tract, then the entire cerebral cortex would not have been activated. Thus, the present data, together with existing information indicate that the desynchronization of EEG or depolarized membrane potential may be an effective indicator of pain during anesthesia.

Traditional NSAIDs such as acetylsalicylic acid nonspecifically inhibit both COX-1 and COX-2. Parecoxib on the other hand, is a new selective COX-2 inhibitor, able to maintain analgesic effects without altering the homeostatic functions of COX-1 [11,19]. In addition, parecoxib is injected and is therefore suitable for patients who cannot take oral medication. In this study, we systematically compared the analgesic effects of parecoxib and fentanyl through electrophysiological techniques. We demonstrated that fentanyl successfully inhibited the electrophysiological profile evoked by nociceptive stimulation, and this inhibition was reversed by naloxone. Parecoxib, on the other hand, had no effect. Recent studies have demonstrated that parecoxib is effective in treating postoperative pain [20,21]. In addition, the analgesic effects of parecoxib have been demonstrated through fMRI studies showing a reduction in acute mechanical pain and mechanical hyperalgesia induced brain activity [22]. Moreover, other reports on postoperative analgesic effects of parecoxib, have found a decrease in pain score and in opioid consumption [20]. There are a number of possible reasons for the negative results in our study. First, surgery injury leads to inflammation and COX-2 is highly expressed in injured tissue, while acute mechanical stimuli (used in this study) only activates the A_δ _and C fibers without abnormally high expression of COX-2. Our results are in line with the results of recent studies reporting no improvement of postoperative pain management after discectomy with parecoxib [23]. These studies found that the timing of parecoxib administration is vital to its effectiveness: peri-operative administration was more effective than postoperative administration [23,24]. Another study found that parecoxib inhibited behavioral changes seen in carrageenan evoked inflammation, but had no effect in acute pain models such as acetic acid-induced writhing and the formalin test [25]. COX-2 mRNA and protein in spinal cord was increased for 3-6 h after injection of carrageenan [26], while acetic acid and formalin induced increases in COX-2 mRNA and protein lasted less than 60 min. In our study, the analgesic effect of parecoxib was tested 20 min after administration when there may not have been abnormally high levels of COX-2 expressed. Therefore, it is possible that the negative parecoxib findings in the present study may be due timing of drug administration. Our results, together with the previous findings suggest that the first dose of parecoxib should be administered before or during a procedure, and that treatment should continue post operation. Our study also suggests that parecoxib should not be used for analgesia in mechanical pain without inflammation.

The inhibitory effect of opioids on dorsal horn projection neurons has been well studied. For example, morphine can inhibit voltage-gated calcium channels of dorsal root ganglion neurons [27]. In addition, topical spinal application of 10 μM morphine produces an inhibitory effect on projection neurons of lumbar spinal cord [28,29]. Previous electrophysiological studies have suggested that ACC pyramidal cells undergo rapid and prolonged depolarization after the induction of acute pain, such as by digit amputation [30]. It is conceivable that cortical desynchronization induced by mechanical stimulation results in the enhancement Ca^2+ ^of influx through voltage-gated Ca^2+ ^channels and NMDA receptors. Opioid agonists were shown to inhibit voltage-gated Ca^2+ ^currents in neurons and reduce excitation by synaptic inhibition [31]. Recent study has shown that low doses of fentanyl could block the induction of LTP in rat spinal cord in vivo [32], which can be explained by the action of opioid receptors on synaptic transmission: presynaptic inhibition of Ca^2+ ^channels and membrane hyperpolarization by activation K^+ ^channels [31]. So the cortical desynchronization might also be blocked by the above mechanisms of fentanyl. In addition, glutamatergic transmission can also be inhibited by opioid agonist, for example, morphine intra-basolateral amygdaloid nucleus injection reduces pain-induced aversion [33]. It is well known that, fentanyl has significant side effects, including respiratory depression, which may confound its analgesic effects [9]. We therefore monitored heart rate and respiratory rate to exclude the possibility of these effects as confounding variable. The present results show that heart and respiratory rates were not greatly affected by fentanyl administration (Fig. 7), and there was no affect on blood gas analysis (table 1). However, there was a slight respiratory depression with fetanyl administration, possibly leading to disruption in internal homeostasis [9]. In the future it will be necessary to exclude the influence of respiratory depression on fentanyl's analgesic effects.

Synaptic and cellular mechanisms of LTP in the ACC are well studied, which is believed to potentiate the sensory responses after injury. Field recording and whole cell patch-clamp recording techniques have been used to study the LTP of excitatory synapse responses in the ACC [34,35]. NMDA receptors play an important role in prefrontal cortex including ACC LTP [34], while NR2A and NR2B subunits contribute to most of the NMDA receptor currents [35,36]. Gene knockout and pharmacological methods have shown that calcium-activated adenylyl cyclases, especially subtype 1 and 8 (AC1, AC8), contribute to the induction of LTP in the ACC [37]. In addition, activity-dependent immediate early genes such as Egr1 and CREB (Cyclic-AMP Response Element Binding proteins) are suggested to contribute to synaptic plasticity [38]. Furthermore, the enhanced synaptic responses persist for a long period in the ACC neurons after tissue inflammation or amputation [34,39]. The animal model of neuropathic pain shows that postsynaptic AMPA receptor mediated responses are enhanced in the ACC synapses, such nerve injury triggered enhancement of postsynaptic responses could be abolished in AC1 knockout mice [40], suggesting that AC1 may serve as a potential therapeutic target for treating neuropathic pain. Recent research has examined potential changes in NR2B receptors in the ACC with inflammatory pain, which showed that NR2B receptor mediated responses are enhanced after peripheral inflammation. With selective forebrain NMDA NR2B overexpression in the case of tissue injury and inflammation, the inflammatory pain and allodynia were significantly enhanced [39,41], providing evidence that NMDA NR2B receptor is critical for chronic pain. Changes in NMDA NR2B mediated EPSCs by nerve injury could also abolished in AC1 lacking mice. ACC is a critical structure for generating emotional response and encoding pain affects [42,43]. Short and long-term plasticities of ACC synapses have been believed to be the cellular basis for the development of chronic pain [44,45]. Lesions of the ACC in patients and inhibition of NMDA receptor/cAMP pathways in the ACC have been shown to be analgesic in chronic pain conditions [46], providing key basic evidence for future chronic pain treatment.

## Conclusion

The cortical activity of the ACC and S1 is desynchronized during noxious mechanical stimulation, while the neural membrane potential is locked in a persistent Up state. Our study provides a useful model for the detailed study of the electrophysiology of the cortex under different states. The activated cortical response to nociceptive stimulation in our study provides a measure for monitoring analgesia during surgery. The results suggest that desynchronization of EEG recordings during surgery should be avoided. Although parecoxib was not effective in inhibiting the cortical response to acute pain in our study, other studies with an alternative dosing schedule should be conducted.

## Materials and methods

### Animal preparation

Adult Sprague-Dawley rats weighing 240-330 g were group housed, 5 per cage and maintained under a 12-h dark-light cycle at 22 ± 1°C. Food and water were available ad libitum. All procedures complied with the Animal Use Committee of Shanghai Institutes for Biological Sciences, Chinese Academy of Sciences. Following initial anesthesia with urethane (1.5 g/kg, i.p.), animals were mounted on a stereotaxic apparatus. Lidocaine was applied locally to the scalp prior to surgery. A sufficient level of anesthesia was maintained throughout surgery such that animals had no spontaneous whisker or eyelid movement. A ceramic pressure sensor was used to monitor respiration and heart rate. Rectal temperature was maintained at 37-38°C through an auto feedback electrical heating blanket. Supplementary doses (one-fifth of initial dose) of urethane were given when necessary. Small craniotomies were made for microelectrode implantation. According to the rat atlas of Paxinos and Waston [47], stereotaxic coordinates were: (1) for SI, 2.5 mm posterior to Bregma (-2.5 A) and 5.0 mm lateral to midline (L); (2) for ACC, 2.0 A, 0.8 L and 1.0-3.0 mm ventral to the brain surface; (3) for V1, -7.0 A, 3.0 L; (4) for PtA, -4.5 A, 4.0 L; and FrA, -5.0 A, 2.0 L. A 2.0 mm diameter incision was made at the corresponding areas by dental drill, and epidural mater was removed to expose the cortex.

### Experimental procedure

For extracellular recordings a tungsten microelectrode (impedance of approximately 450 KΩ) was slowly lowered into the ACC with a microdriver (Narishige) under a dissecting microscope while a reference electrode was placed just below the scalp. The extracellular signal (0.1 to 3000 Hz) was acquired at a sampling frequency of 20 KHz and then passed through an amplifier (MODEL 2000, A-M system), digitalized by a MICRO 1401 A/D converter (Cambridge Electronic Design) and then stored with Spike 2 software (Cambridge Electronic Design).

Intracellular recordings from barrel cortical neurons were performed by sharp glass microelectrodes pulled from glass capillaries with filament (outer diameter of 1.5 mm). The electrode (filled with 2 M potassium acetate) had a tip resistance of 60-100 MΩ. After removing the epidural mater, the electrode was advanced to the surface of brain tissue with a motorized manipulator (Narishige) under a dissecting microscope. The cortical exposure was sealed with low-melting agar to reduce fluctuation of brain tissue when the electrode was lowered into the brain. The electrode was slowly advanced with the microdriver at 3-5 μm steps. Intracellular recordings began after ACC extracellular activity was enhanced by tail pinch. The electrical signal was amplified by an AxoClamp2B amplifier (Molecular Devices), converted by a MICRO 1401 and stored for offline analysis. Data from neurons with a resting membrane potential below -60 mV and spontaneous spike amplitude > 50 mV were included in the study.

After acquiring a stable extracellular and intracellular recording, a neuronal response was elicited by pinching the tail using small forceps (constant force: ~350 g/mm^2^) for about 15 s. The stimulus was applied 2 or 3 times with an inter-stimulus interval of 5 min. The effect of parecoxib (20 mg/kg, i.m.) or fentanyl (30 μg/kg, i.m.) on the mechanical stimulation induced response was tested, and repeated stimulus was applied 20 min or 5 min after each drug injection, respectively. Naloxone (30 μg/kg, i.m.) was used to reverse fentanyl's action. In addition, abdominal aorta blood was collected and measured for blood gas analysis from eight rats before or 5-10 min after fentanyl injection.

### Data analysis

Raw signals were visually inspected and artifacts were deleted before analysis. MATLAB (Mathworks,), OriginLab 8 (OriginLab) and Spike 2 software were used for data analysis. FFT (fast Fourier transform, Hanning window) was performed on the EEG data (filtered out at 1-100 Hz) to obtain a power spectrum from which we calculated the integrations of power within the following bands [48]: total, 1-100 Hz; δ, 1-4 Hz; θ, 4-8 Hz; α, 8-12 Hz; β, 12-25 Hz; γ, 25-100 Hz. For a more specific description, we plotted the cumulative power distribution (CPD) curve based on the power spectrum by directly acquiring MF and SEF (frequency below which 50% and 95% of total power resides, respectively). We then measured the "distance" of cumulative power distribution, defined by the area of the region between any two given cumulative curves. This measure quantifies the change of power distribution under two corresponding experimental conditions. A similar analysis was used in describing the change of the membrane potential distribution from intracellular recordings.

Multi-unit data were extracted from the extracellular signal through a band-pass filter (600~3000 Hz), and appropriate thresholds were manually selected for spike discrimination. To describe the uniformity of the spike train in the absence and the presence of the nociceptive stimulus, we defined a "Non-uniform Index" of any given time period *T *with a spike number of *N *as

where *t*_i _stands for the time of the ith spike. This index adequately caught the distinct characteristics of the "Up and Down" in contrast to the persistent "Up" firing patterns, regardless of their overall mean firing rates.

To collect data from intracellular recordings, we first distinguished the Up and Down states by comparing the average membrane potential during a given time window (~10 ms) with that of a much larger time span (~3 s). We then counted the frequency of membrane potential transitions between the two states. In addition, as described above for extracellular recording analysis, the distance between two cumulative curves was used to quantify differences between experimental groups.

### Statistical analysis

Data were normally distributed and presented as mean ± SEM. Statistical significance was tested with paired *t-*tests using p < 0.05 as statistically significant.

## List of abbreviations

EEG: electroencephalogram; S1: primary somatosensory cortex; S2: secondary somatosensory cortex; ACC: anterior cingulate cortex; LTP: long-term potentiation; NSAIDs: anti-inflammatory drugs; COX-2: cyclooxygenase-2; PtA: parietal association cortex; V1: primary visual cortex; FrA: frontal association cortex; CPD: cumulative power distribution; MF: medial frequency; SEF: 95% spectral edge frequency; MU: Multi-unit; V_m_: membrane potential; AMPA: α-amino-3-hydroxyl-5-methyl-4-isoxazole-propionate; NMDA: N-methyl-D-aspartic acid.

## Competing interests

The authors declare that they have no competing interests.

## Authors' contributions

YZP participated in the design of the study, carried out the experiment, and drafted the manuscript. XXL assisted with the electrophysiological recording. YWW conceived of the study, assisted with the data analysis, and wrote the manuscript. All authors read and approved the final manuscript.
